# The 18 kDa Translocator Protein (Peripheral Benzodiazepine Receptor) Expression in the Bone of Normal, Osteoprotegerin or Low Calcium Diet Treated Mice

**DOI:** 10.1371/journal.pone.0030623

**Published:** 2012-01-25

**Authors:** Winnie Wai-Ying Kam, Steven R. Meikle, Colin R. Dunstan, Richard B. Banati

**Affiliations:** 1 Discipline of Medical Radiation Sciences, Faculty of Health Sciences, University of Sydney, Cumberland, New South Wales, Australia; 2 Ramaciotti Imaging Center, Brain and Mind Research Institute, University of Sydney, Camperdown, New South Wales, Australia; 3 Bone Research Program, Anzac Research Institute, University of Sydney, Concord, New South Wales, Australia; 4 Australian Nuclear Science and Technology Organisation, Lucas Heights, New South Wales, Australia; 5 School of Aerospace, Mechanical and Mechatronic Engineering, University of Sydney, Camperdown, New South Wales, Australia; Stanford, United States of America

## Abstract

The presence of the translocator protein (TSPO), previously named as the mitochondrial or peripheral benzodiazepine receptor, in bone cells was studied *in vitro* and *in situ* using RT-qPCR, and receptor autoradiography using the selective TSPO ligand PK11195.

*In vitro*, the TSPO is highly expressed in osteoblastic and osteoclastic cells.

*In situ*, constitutive expression of TSPO is found in bone marrow and trabecular bone, e.g., spongiosa. Mice with a reduction of bone turnover induced by a 4-day treatment of osteoprotegerin reduces [^3^H]PK11195 binding in the spongiosa (320±128 Bq.mg^−1^, 499±106 Bq.mg^−1^ in saline-treated controls). In contrast, mice with an increase in bone turnover caused by a 4-day low calcium diet increases [^3^H]PK11195 binding in the spongiosa (615±90 Bq.mg^−1^).

Further, our study includes technical feasibility data on [^18^F]fluoride microPET imaging of rodent bone with altered turnover. Despite [^18^F]fluoride having high uptake, the *in vivo* signal differences were small. Using a phantom model, we describe the spillover effect and partial volume loss that affect the quantitative microPET imaging of the small bone structures in experimental mouse models.

In summary, we demonstrate the expression of TSPO in small rodent bone tissues, including osteoblasts and osteoclasts. A trend increase in TSPO expression was observed in the spongiosa from low to high bone turnover conditions. However, despite the potential utility of TSPO expression as an *in vivo* biomarker of bone turnover in experimental rodent models, our small animal PET imaging data using [^18^F]fluoride show that even under the condition of a good biological signal-to-noise ratio and high tracer uptake, the currently achievable instrument sensitivity and spatial resolution is unlikely to be sufficient to detect subtle differences in small structures, such as mouse bone.

## Introduction

The peripheral or mitochondrial benzodiazepine receptor [1], [2], [3], [4], also referred to as the translocator protein (TSPO) [5], is an 18kDa intracellular protein expressed in many tissues and tends to be up-regulated in conditions of immune stimulation, stress responses as well as increased cell turnover and tissue damage [2], [4], [6], [7]. The latter most likely relates to the prominent expression of the TSPO in cells of mononuclear phagocyte lineage [7], [8]. While the TSPO expression has been found in human osteoblast cultures [9], its expression in osteoclasts or in bone tissue in differing states of turnover has not yet been reported.

There is broader interest in the TSPO as an *in vivo* imaging marker of active tissue pathology. One well characterised TSPO-selective radioligands suitable for positron emission tomography (PET) is 1-(2-chlorophenyl)-*N*-methyl-*N*-(1-methylpropyl)-1-isoquinoline carboxamide ([^11^C]PK11195) [10], [11]. It has been used to image active tissue pathology in the human central nervous system, by virtue of the prominent expression of TSPO in activated microglia, the brain intrinsic macrophage population [12], [13], [14], [15].

In this study, we examined the expression of TSPO in mouse primary bone cultures and bone tissues with altered turnover rates. In addition, we investigated the feasibility of microPET, i.e. small animal positron emission tomography, to quantify altered bone turnover *in vivo* using [^18^F]fluoride as a benchmark radiotracer with high uptake and good signal-noise ratio.

## Materials and Methods

Animal ethical approval was obtained for the experiments reported in this study: Approved by the South West Area Health Service Area Health Service Animal Welfare Committee - Approval number 2005/010A (Title: Bone Imaging in rats and mice by micro-PET) and by the University of Sydney Animal Ethics Committee – Approval number A41/11-2005/3/4196 (Title: Bone Imaging in rats and mice by micro-PET).

### Mouse models of altered bone turnover

Five-week old CD-1 mice were maintained on a normal diet for 1 week prior to any treatment. At this time, the mice were randomized on Day 0 into low bone turnover, high bone turnover or normal groups.

Low bone turnover was produced in mice on a normal diet by administering a single subcutaneous injection (5 mg/kg on Day 0) of a recombinant construct of osteoprotegerin (OPG) containing amino acids 22–194 of human OPG fused to the Fc domain of human immunoglobin G (Fc-OPG) (kindly provided by Amgen, Inc., Thousand Oaks, CA, U.S.A.). This dose of long-acting Fc-OPG has been previously shown to inhibit bone resorption profoundly and to reduce bone turnover for this period of study [16].

High bone turnover was induced using dietary calcium restriction as previously reported [17]. In brief, mice were provided with a commercially-available low calcium diet (Specialty Feeds, Glen Forrest, WA, Australia) from Day 0 – calcium content was reduced to 0.1 g/100 g whereas the calcium content in the normal mouse diet was 0.6 g/100 g. The diet was in the form of 12 mm diameter pellets. These mice also received a subcutaneous injection of saline on Day 0 to assure direct comparability with the low bone turnover mice.

Control mice were mice on a normal diet with subcutaneous injection of saline on Day 0.

On day 4, the treated mice were used for microPET study (mice with OPG or low calcium diet treatment only, N = 4/group), or were euthanized by cervical dislocation either for film autoradiography study (all 3 types of mouse models, N = 4/group) or RNA extraction and RT-qPCR (1 control mouse).

### Primary osteoblast and osteoclast culture

Primary osteoblastic cells were prepared from the calvaria of newborn CD-1 mice by digestion with 0.1% collagenase (Worthington Biomedical Co., Lakewood, NJ, U.S.A.) and 0.2% dispase (Invitrogen, Carlsbad, CA, U.S.A.). Cells were cultured in 24-well plates at a density of 10^5^ cells/well in alpha-minimum essential medium (áMEM; Invitrogen, Carlsbad, CA, U.S.A.) containing 10% fetal bovine serum (FBS). Cells were allowed to attach for 24 hours, and then media were replaced with áMEM containing 10% FBS, ascorbic acid (50 µg/ml; Sigma Aldrich) and â-glycerophosphate (10 mM; Sigma Aldrich). Cells were cultured for a further 7 days with fresh medium replacement every 2 days.

Mouse spleen cells were obtained from 6 week-old CD-1 mice and cultured for 7 days in áMEM containing 10% FBS, 25 ng/ml macrophage colony-stimulating factor (M-CSF; R&D Systems, Minneapolis, MN, U.S.A.), and 50 ng/ml receptor activator of nuclear factor κβ ligand (RANKL) (R&D Systems, Minneapolis, MN, U.S.A.) for 7 days with fresh medium replacement every 2 days.

### RNA isolation, reverse transcription and real-time PCR

The femurs and tibias of 1 control mouse were harvested. After the muscles were carefully removed, the bone tissues (∼100 mg each) were snap frozen in liquid nitrogen then used immediately for RNA extraction. Total RNA was isolated from mouse bone tissue using TRIzol reagent (Invitrogen, Carlsbad, CA, U.S.A.), or from cultured osteoblasts and osteoclasts using NucleoSpin® RNA II Kit (Macherey-Nagel, Düren, Germany) following the manufacturers' protocols. TSPO mRNA expression has been reported in skeletal muscle [18], thus RNA extraction was also performed for some of the mouse skeletal muscles which were to be used as positive control in the present study. The concentration of the RNA was determined using the BioPhotometer (Eppendorf, Hamburg, Germany). The purity of the extracted RNA was assessed spectrophotometrically using the A260/A280 ratio, while RNA integrity was assessed by the 28s/18s ratio after electrophoresis on 1% agarose gel.

First strand cDNA was synthesized from 2 µg of total RNA using the SuperScript® III First-Strand Synthesis Super Mix kit (Invitrogen, Carlsbad, CA, U.S.A.) according to the manufacturer's instruction. The freshly prepared cDNA was further diluted by DEPC-treated water and the subsequent real-time PCR was performed by CFX 384™ Real-Time PCR Detection System using SsoFast supermix (BioRad, California, U.S.A.). Bone markers i.e. alkaline phosphatase (ALP) for osteoblast [19] and tartrate resistant acid phosphatase (TRAP) for osteoclast [20], as well as TSPO mRNA expression levels in the above primary cell cultures and bone tissues were investigated (For PCR primer sequences, see Table S1). The annealing temperature was empirically optimized so as to accommodate all the PCR primers in a single run. The PCR conditions were 98°C for 30 seconds, followed by 45 cycles at 98°C for 5 seconds, 60°C for 10 seconds. At the end of the 45^th^ cycle, the temperature was raised to 72°C for 10 minutes to ensure complete extension of the PCR products. A melt curve analysis was performed after the PCR to confirm the specificity of the results. Another PCR without the melt curve analysis was performed using TSPO primer on the whole bone tissue only and the PCR product was used for restriction enzyme assay (see below).

Glyceraldehyde 3-phosphate dehydrogenase (GAPDH) [21] (Table S1) was used as internal standard. The relative expressions of target genes were quantified using comparative C_t_ analysis incorporated in the CFX Manager™ Software (version 1.5) (BioRad, California, U.S.A.).

### Restriction enzyme assays and DNA sequencing

Two types of restriction enzymes were used for digesting the PCR products amplified from the TSPO primers: (1) the blunt ends PvuII (BioLabs Inc., Ipswich, MA, U.S.A.), cutting at nucleotide position 202; (2) the sticky ends NcoI (BioLabs Inc., Ipswich, MA, U.S.A.), cutting site at 114/118. Digestion was performed for 1.5 hours at 37°C. The digests were visualized on 1.5% agarose gel by ethidium-bromide staining.

In order to further confirm the specificity of the results, some of the undigested PCR products were purified using the purification kit NucleoSpin ® Extract II (Macherey-Nagel Inc., Bethlehem, PA, U.S.A.) and sent to the Sydney University Prince Alfred Macromolecular Analysis Centre for DNA sequencing.

### Film autoradiography using [^3^H]PK11195

The femurs from the control mice and mice with high or low bone turnover (N = 4/group) were decalcified in 10% EDTA for 7 days before tissue sectioning. Cryostat bone sections (10 µm) were placed onto superfrost slides (Menzel-Glaser, Braunschweig, Germany) and stored in the dark at −20°C for no longer than 1 week before the experiment. The film autoradiography was performed according to the established method [12], [22] using R-[N-methyl-^3^H] PK11195 ethanol solution, with a specific activity of 3.14 TBq.mmol^−1^ (PerkinElmer, Waltham, Massachusetts, U.S.A.). Tritium standards (Amersham Biosciences, Uppsala, Sweden) co-exposed with the bone sections on each Hyperfilm-^3^H (Amersham Biosciences, Uppsala, Sweden) at 4°C for 14 days, were used to quantify the autoradiographically measured binding.

The hyperfilm was developed using Kodak GBX developer and fixer (Sigma Aldrich, St. Louis, MO, U.S.A.). The hyperfilm was air-dried overnight before being scanned using the ArtixScan 1800f flatbed scanner (Microtek, Hsinchu, Taiwan), with greyscale using a magnification of 400% and an optical resolution of 2400 pixels per inch. No pre-scanning manipulation or filter was applied.

**Figure 1:**
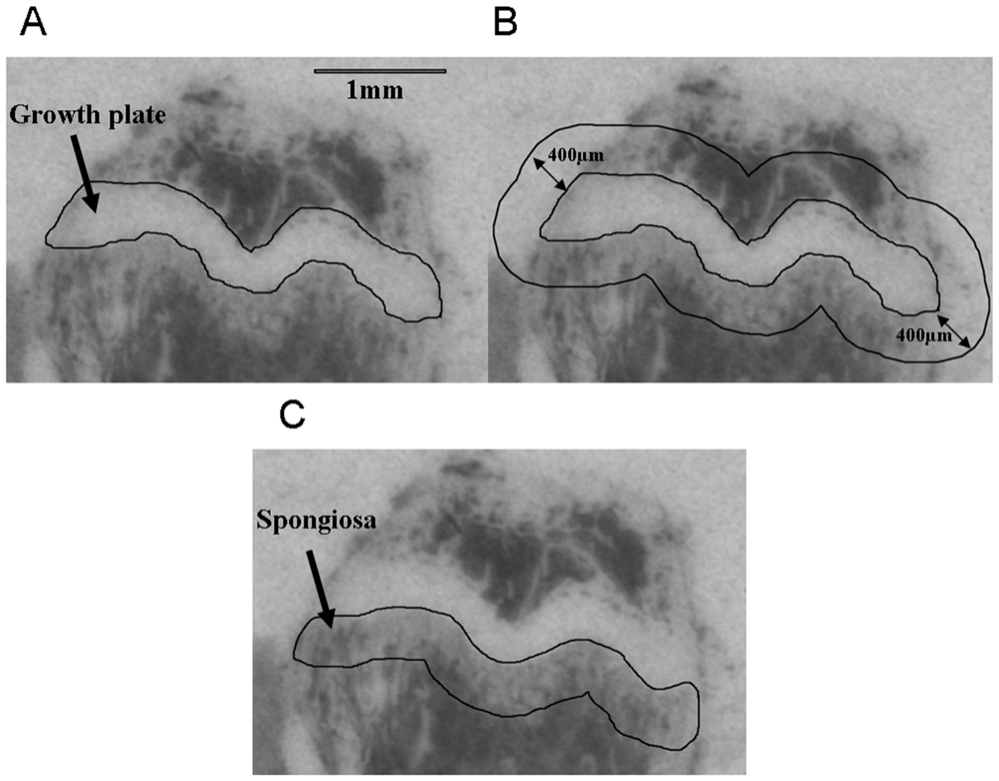
Steps for defining the sub-growth plate spongiosa as the ROI in a bone autoradiograph. (A) The growth plate is readily identified as a landmark. (B) shows the consistent expansion of 400 µm around the initially defined landmark structure of the growth plate (C) and the resultant delineation of the sub-growth plate spongiosa.

The film-autoradiographic quantification of [^3^H]PK11195 binding indicating regional TSPO receptor expression was performed on the scanned hyperfilms using ImageJ (freeware by Wayne Rasband, National Institutes of Health, U.S.A.) without further image processing, such as contrast variation or background subtraction. On film-autoradiographic images of the bone, the growth plate imposes as a readily identifiable structure of high contracts. It can, therefore, be used as a robust anatomical landmark to place a region of interest (ROI) for quantification of the level of receptor binding in a defined area of the spongiosa of the bone. The ROI, i.e. the spongiosa, which is directly adjacent to the growth plate, was defined by a semi-automated process consistently applied to all samples as shown in Figure 1. It involves as the only manual procedure the delineation of the anatomical landmark (Figure 1A), i.e. the growth plate to serve as the kernel expansion of a 400 µm perimeter around the landmark outline using the “Enlarge” tool (Figure 1B) and then the measurement of the inverted grey values from 6 to 7 serially cut bone sections in the isolated sub-growth plate spongiosa (Figure 1C). Grey values were converted into Bq/mg using the tritium standards (Amersham Biosciences, Uppsala, Sweden). Statistical analysis of intergroup variation was carried out using one-way ANOVA followed by Tukey's multiple comparison test, and deemed significant at p<0.05.

### MicroPET study

Dynamic skeletal scans were performed using the microPET® Focus™ 220 animal scanner (Preclinical Solutions, Siemens Healthcare Molecular Imaging, Knoxville, TN, USA). This device has a 7.6 cm axial field of view (FOV), 19 cm transaxial FOV and resolution at the centre of the FOV of 1.4 mm. The mouse was placed on a heating pad (38°C) to acclimatize for 20 minutes prior to anesthetizing with 2% isoflurane in oxygen. With the animal in a supine position, the hind legs of the mouse were spread alongside of its body, with the tibia parallel to the long axis of the animal. A pressure transducer was taped to its chest for monitoring respiration throughout the scan.

No carrier added-aqueous [^18^F]fluoride ion produced on a PETtrace cyclotron (GE Healthcare, Sweden), was diluted to a concentration of approximately 30 MBq in 0.3 mL saline and injected via the tail vein of the mouse for an 1-hour dynamic emission scan (with a ring difference of 47 and span of 3). An energy window of 350 to 650 keV and coincidence timing window of 6 nanoseconds were used in all studies. No transmission scan was performed and no attenuation or scatter correction was applied to the data. Dead time correction, arc correction and decay correction were applied to the acquired data prior to image reconstruction.

**Figure 2:**
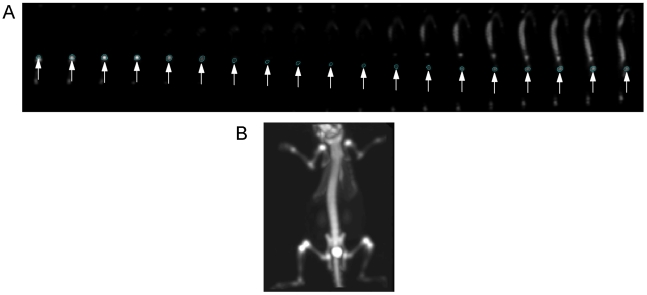
MicroPET tomogram of a mouse skeleton. (A) shows the placement of the ROI on femur bone in the sagittal plane where it is a clearly delineated circular area (indicated by white arrows). The elongated structure in the last 8 planes of the image is the spinal column. (B) shows a coronal plane of an OPG-treated mouse microPET tomogram. The bright spot at the pelvic level is the bladder.

Post-processing and viewing of the reconstructed images were performed using the supplied microPET software – microPET ASIPro VM™ 6.2.1.8 (ASIPro; Siemens Medical Solutions U.S.A., Inc.). The 3D sinograms were reconstructed using the MAP algorithm [23] with 18 iterations and a β value of 0.0001. 3D ROIs were drawn manually over the femur along the sagittal planes (Figure 2A; a coronal plane of a mouse microPET tomogram is shown in Figure 2B) of the summed image of each animal for standardized uptake values (SUVs) quantification i.e. tissue activity uptake normalized by the injected dose per unit weight [24]. Statistical analysis of intergroup variation (only mice with OPG treatment and mice on a low calcium diet were used in this experiment) was carried out using Student's t-test, and deemed significant at p<0.05.

### Phantom design, construction and data acquisition

**Figure 3:**
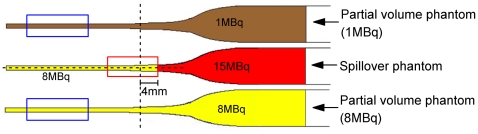
The gelatin phantom to simulate partial volume loss and spillover effect *in vivo*. The dashed line represents the centre of the microPET axial field of view and is positioned 4 mm from the interface between the region containing 8 MBq and the region containing 15 MBq of activity. The red box shows the region affected by spillover effect. The blue boxes are the areas affected by partial volume losses due to the small volume of the structure.

For simulating a mouse femur in an *in vivo* situation, a phantom set was prepared using glass Pasteur pipettes filled with gelatin (20% of 20N Grade edible gelatine; GELTA Australia Pty Ltd) containing different levels of radioactivity (Figure 3): (1) the partial volume phantom (1 MBq) containing 1 MBq of [^18^F]fluoride; (2) the partial volume phantom (8 MBq) containing 8 MBq of [^18^F]fluoride; and (3) the spillover phantom containing 8 MBq of [^18^F]fluoride with an additional 15 MBq of [^18^F]fluoride on top. A 360-second static emission scan was acquired of the phantoms. The phantoms were located in the scanner such that the centre of the field of view was positioned 4 mm from the interface between the regions of 8 and 15 MBq of [^18^F]fluoride (Figure 3). The same acquisition settings and reconstruction parameters as in the animal study described above were used. For post-processing, a fixed circular 2D ROI (4 mm×4 mm) was drawn on each transverse plane of the MAP reconstructed image along the long axis of each glass Pasteur pipette. The maximum ROI value was calculated and plotted against the plane number to produce an activity profile.

## Results and Discussion

### TSPO mRNA expression in whole bone, osteoclasts and osteoblasts

**Figure 4:**
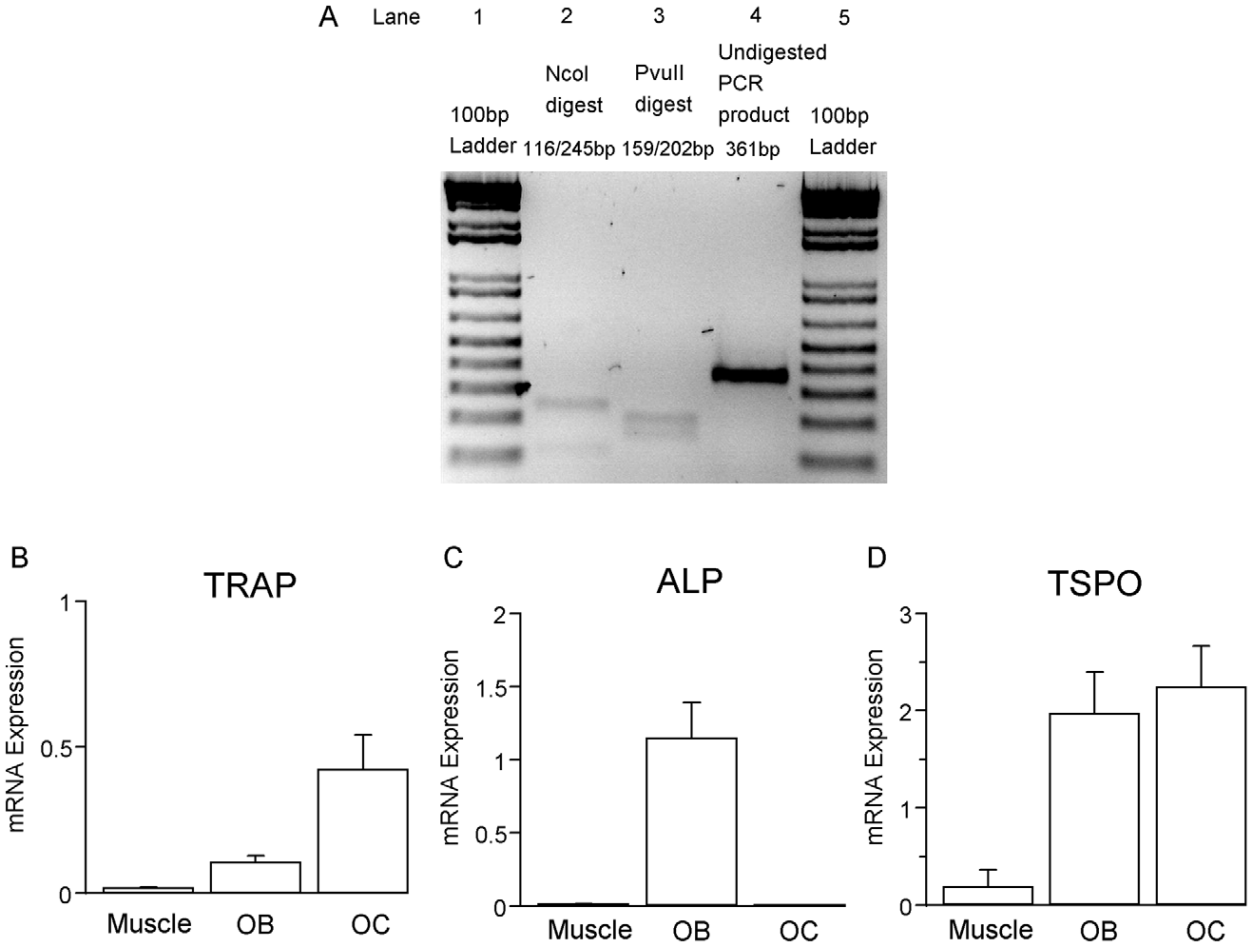
TSPO mRNA expression in bone. (A) Lanes 1 and 5 show 100 bp DNA ladders. TSPO mRNA is detected in the whole bone tissue of a normal mouse (lane 4). Restriction enzyme assay was used to confirm the specificity of the result. PCR products of expected band sizes are produced from the digestion by NcoI (lane 2) and PvuII (lane 3). (B) shows the expressions of TRAP, (C) the expression of ALP and (D) the expression of TSPO mRNA in primary osteoclast and osteoblast cultures and in skeletal muscle (N = 3). OB = primary osteoblast culture from calvaria; OC = primary osteoclast culture from spleen cells treated with M-CSF/RANKL. Error bar = standard deviation.

The TSPO primers used in the current study demonstrated high specificity to the target gene. A single band of the expected size (361 bp) was amplified from the whole bone tissue of a normal mouse (Figure 4A, lane 4). The specificity of the product was confirmed by restriction enzyme assay. PCR products amplified from the whole bone tissue were digested using the selected restriction enzymes, yielding products of the predicted band sizes, i.e. NcoI yielded products of 116 bp and 245 bp (Figure 4A, lane 2); while PvuII digestion yielded products of 159 bp and 202 bp (Figure 4A, lane 3). Furthermore, sequencing of the amplicon confirmed the gene as mouse TSPO (Genbank accession number NM_009775).

RNA isolated from whole bone demonstrated significant TSPO gene expression. Since hematopoietic cells, such as monocytes [25], polymorphonuclear neutrophils [26], lymphocytes [27], platelets [11], [28] and erythrocytes [29], too, express TSPO, it is likely that some of the TSPO gene expression found in whole bone is due to contaminating bone marrow which is difficult to fully remove from a mouse femur of 1 mm diameter. Therefore, pure primary osteoclasts derived from mouse spleen cells with M-CSF/RANKL treatment and osteoblasts derived from newborn mouse calvaria were examined for the presence of TSPO gene products. Using TRAP and ALP as cell-type specific markers for osteoclasts and osteoblasts, respectively, revealed that both primary TRAP-expressing osteoclasts (Figure 4B) and ALP-expressing primary osteoblasts (Figure 4C) express the TSPO gene (Figure 4D). Primary osteoblasts and osteoclasts had similarly high TSPO mRNA expression levels that were approximately 10 times higher than that in skeletal muscle [18] (Figure 4D).

Our finding of TSPO expression in primary osteoblasts is consistent with a previous report for human osteoblasts [9]. In addition, we found that TSPO mRNA is also expressed in osteoclastic cells (Figure 4D). This TSPO expression is comparable to that seen in microglia and macrophages, that like osteoclasts are of monocyte/phagocyte lineage [30], [31].

### Regional [^3^H]PK11195 binding in normal mouse bone

The selective ligand PK11195 binds to the TSPO with a single high affinity [32] and, labelled with the positron-emitter [^11^C], has been used to image neuroinflammatory brain pathology *in vivo* by PET [12], [14]. Here, we used the tritiated ligand [^3^H]PK11195 and film autogradiography [12], [22] to complement TSPO mRNA expression data and show the regional expression of the TSPO receptor in bone tissue, more specifically the highly dynamic sub-growth plate spongiosa.

**Figure 5:**
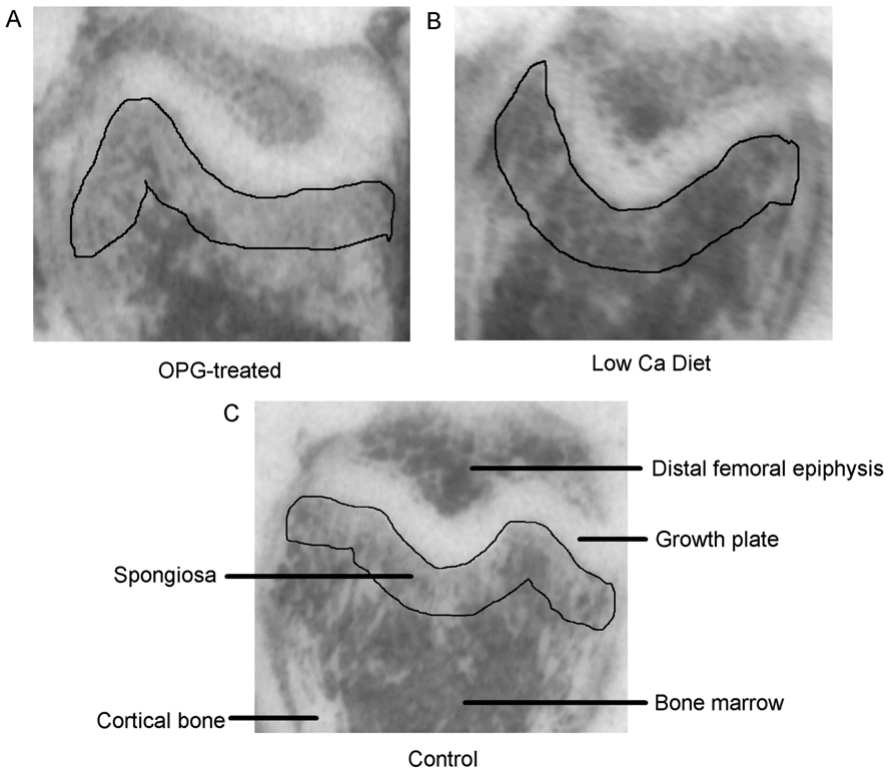
[^3^H]PK11195 film autoradiography of bone. [^3^H]PK11195 binding in the spongiosa (sub-growth plate region) is lower in mice treated with OPG (A), compared to mice on a low calcium diet (B) and at an intermediate level in saline-treated control mice (C).

Figure 5 shows the autoradiographs of the mouse femur from each treatment group. Dark areas of the section, representing high levels of bound [^3^H]PK11195 were observed beneath the growth plate, i.e. in the primary and secondary spongiosa (area enclosed by the black line), and in the trabecular bone of the epiphysis. No or negligible [^3^H]PK11195 binding was detected in the cortical bone or within the growth plate itself.

The bone autoradiographs showed a marked decrease in the trabecular bone architecture in the distal femur of the OPG-treated mice when compared to those from mice with low calcium diet (Figure 5A and B). Control mouse showed an intermediate level (Figure 5C). Spongiosa is mainly composed of trabeculae, which have a greater surface area per unit bone volume than cortical bones. Thus, trabeculae have higher turnover than cortical bone tissue [33], particularly in mice that do not have Haversian remodeling of cortical bone [34]. The observed distribution pattern of [^3^H]PK11195 binding in bone overlaps closely the areas in the bone that are known to have high turnover, such as the sub-growth plate spongiosa [33].

### Regional [^3^H]PK11195 binding levels in mouse models of altered bone turnover

**Figure 6:**
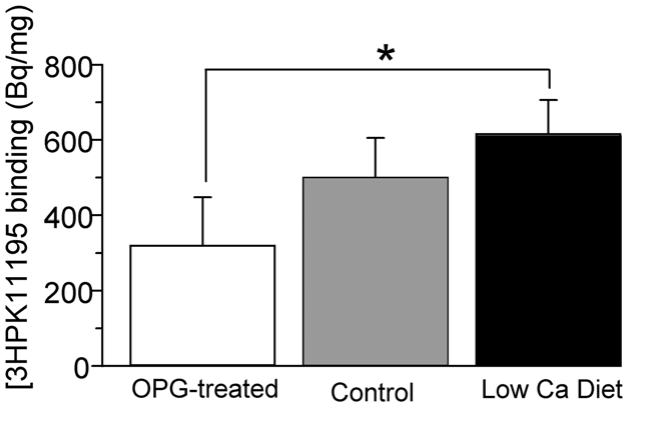
Quantification of [^3^H]PK11195 binding in mice with altered bone turnover. The regional binding in the sub-growth plate spongiosa is quantitatively expressed as tissue equivalent activities (Bq/mg; N = 4/group). A significant difference (*) is observed between animals with OPG and low calcium diet treatments (p = 0.01, one-way ANOVA with Tukey's multiple comparison test). Error bar = standard deviation.

The [^3^H]PK11195 binding levels quantified by high resolution film autoradiography showed differences between the three treatment groups (320±128 Bq.mg^−1^ for the OPG-treated group; 499±106 Bq.mg^−1^ for the saline-treated group and 615±90 Bq.mg^−1^ for the low calcium diet group; p = 0.012; Figure 6). A significant difference was found between the OPG-treated and the low calcium diet mice (p = 0.01, Tukey's multiple comparison test). However, the separation of the treated animals of either kind from the saline-treated control group was too little to reach statistical significance (Tukey's multiple comparison test: OPG-treated mice compared to the saline-treatment p = 0.1; low calcium diet mice compared to the saline-treatment p = 0.33). This suggest that despite the high spatial resolution of approximately 100 µm^2^ [35] achievable with film autoradiography, [^3^H]PK11195 binding is unlikely to be a suitable indicator of pathological bone turnover.

### Feasibility of skeletal microPET imaging – partial volume loss and spillover effect

There are currently few non-invasive methods for measuring bone turnover in experimental rodent models. Despite the general observation that TSPO expression as measured by increased PK11195 binding correlates well with the presence of tissue pathology in many different organs and can also be observed in altered bone turnover, our tissue binding data suggest that the differences in bone measured by [^3^H]PK11195 are small. It, therefore, remains to be shown whether carbon-11 labelled PK11195 provides a better signal-to-noise ratio and may be a feasible radioligand for microPET imaging of bone. In contrast, [^18^F]fluoride is already known to have high uptake in bone and thus give rise to a high signal in microPET. Therefore, in order to establish the capability of microPET to measure changes in bone turnover, we imaged mice femur with differentially altered bone turnover. The femur being a large bone is the best region to study since in thinner bones blood activity and unbound [^18^F]fluoride tend to cause confounding effects [36].

**Figure 7:**
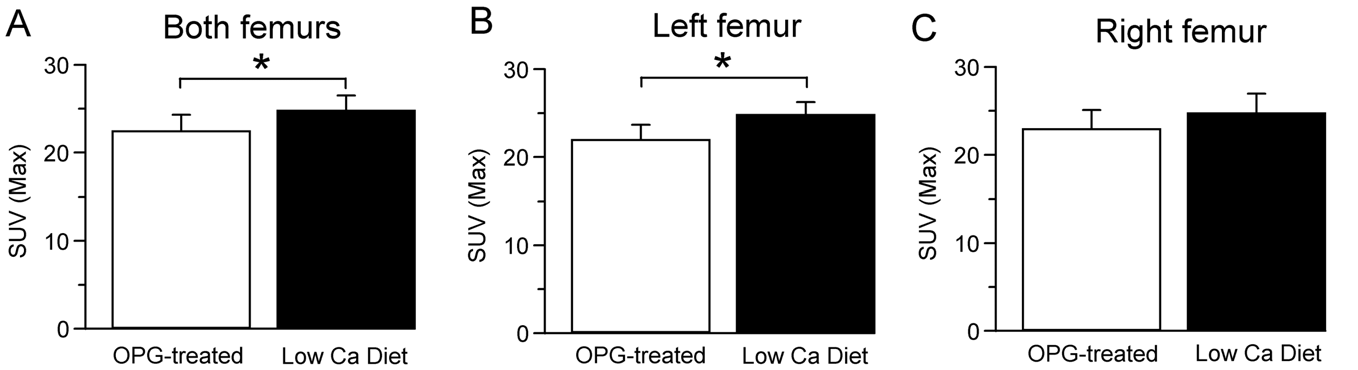
[^18^F]fluoride uptake measured by microPET. [^18^F]fluoride uptake is expressed in SUV for left femur (A), right femur (B) and both femurs together (C) (N = 4/group). Error bar = standard deviation. A significant group difference between OPG-treated and low calcium diet animals can be seen in the pooled bone data. However, the differences are small and not consistent if right and left femurs are compared separately (*right and left femurs*: OPG-treated = 22.4±1.9; low calcium diet = 24.73±1.76, p = 0.023; *left femur*: OPG-treated = 21.91±1.74; low calcium diet = 24.76±1.47, p = 0.046; *right femur*: OPG-treated = 22.89±2.18; low calcium diet = 24.7±2.25, p = 0.293).

In this microPET study, OPG-treated mice were compared to low calcium diet mice, i.e. the groups that have been shown to have the largest difference in bone turnover. The microPET tomogram showed the expected high fluoride uptake in the skeleton with highest uptake in the distal and proximal metaphyses of the main long bones (Figure 2B). These regions correspond to areas of rapid bone growth and probably reflect the rapid mineralization occurring in hypertrophic cartilage of the growth plates and bone formation in the primary and secondary spongiosa. However, microPET using [^18^F]fluoride only revealed subtle and not fully consistent differences between OPG-treated mice and low calcium diet mice (Figure 7) (*right and left femurs*: OPG-treated = 22.4±1.9; low calcium diet = 24.73±1.76, p = 0.023; *left femur*: OPG-treated = 21.91±1.74; low calcium diet = 24.76±1.47, p = 0.046; *right femur*: OPG-treated = 22.89±2.18; low calcium diet = 24.7±2.25, p = 0.293).

**Figure 8:**
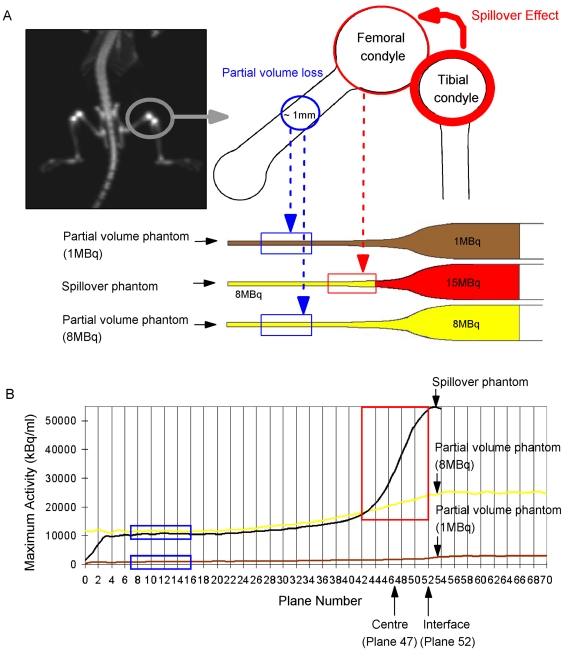
Spillover and partial volume loss phantoms. (A) The coronal [^18^F]fluoride microPET image shows the area of the knee joint where spillover effect and partial volume loss occur (grey circle). The diagram shows how the phantom mimics the spillover effect and partial volume loss. The areas of the phantom that show high partial volume loss are marked by blue boxes; the area of spillover effect is marked by a red box. (B) The activity profiles, plotted from the narrow to the wide end of the glass Pasteur pipette, are shown for each of the phantoms. The red box region (plane numbers 42 to 52) corresponds to the spillover effect region described in (A). The blue boxes (plane numbers 7 to 16) correspond to the partial volume loss regions described in (A). The central plane (plane 47) of the microPET tomogram is indicated in the figure. Plane 52 is the interface between the region of high (15 MBq) and low activity (8 Mbq).

The failure of [^18^F]fluoride-microPET in showing fully consistent differences between animals with low and high bone turnover may have a number of biological and methodological reasons. An obvious reason warranting separate investigation relates to limitations in instrument sensitivity and resolution, which would cause marked spillover effect [37] and partial volume loss [38]. To conclude the feasibility study of [^18^F]fluoride-microPET, a phantom set was designed to replicate the extent to which the spillover effect and partial volume loss suggested by mouse bone microPET datasets are indeed the case (Figure 8).

Spillover effect at the femoral head is likely to be caused by the adjacent tibial head. In our phantom, a high activity gelatin source placed adjacent to a low activity gelatin mimics the effect of the adjacent tibial head (Figure 8A and B – the regions enclosed by the red circles/box). In contrast, partial volume loss is expected to be significant in the region of the mouse mid-femur and is mimicked in our study by the narrow end of a glass Pasteur pipette with a diameter of ∼1 mm, which is the same as a mouse mid-femur (Figure 8A and B – the regions enclosed by the blue circle/boxes), Unlike in the spillover phantom, the activity within the partial volume phantoms is homogenous, i.e. any effects are purely due to variation in volume. The mouse microPET data indicated a ratio of activity at the distal femur to mid-femur of approximately 1 to 8. This activity ratio was reproduced in our phantom (Figure 8A).

The activity profile of our spillover phantom measured by microPET confirmed the presence of significant spillover effect (Figure 8B). Depending on the distance from to the high activity region causing the spillover effect, the actual measured activity in the region of low activity was approximately 4% to 112% higher than the true activities in the phantom (Table 1).

**Table 1:** Spillover effect on a region with low activity.

Reconstruction algorithm	Plane number	Spillover phantom profile (kBq/ml)	Partial volume phantom (8 MBq) profile (kBq/ml)	% increase due to spillover
**MAP**	42	291.7	280.85	3.86
	43	324.26	292.67	10.79
	44	363.28	297.61	22.07
	45	416.2	307.11	35.52
	46	467.12	312.59	49.44
	47	537.21	327.46	64.05
	48	607.56	334.43	81.67
	49	670.57	341.19	96.54
	50	729.26	354.79	105.55
	51	761.89	358.59	112.47
	52	789.88	372.66	111.95

The table shows the plane-by-plane false high measurements due to spillover from a region of high activity (15 MBq) into an area of low activity (8 MBq). The error ranges from approximately 4% to 112% depending on the proximity to the region of high activity.

The activity profiles of our partial volume phantoms (Figure 8B) demonstrated that the measured activity in small ROIs is approximately 55 to 70% below the true activity values (Table 2). Small volumes with low activity show relatively more partial volume loss than small volumes with high activity.

**Table 2:** Partial volume loss in a small region of interest.

	Partial volume phantom (1 MBq) profile	Partial volume phantom (8 MBq) profile
Averaged activity in region of high partial volume loss (Planes 7 to 16) (kBq/ml)	921.52	11600.97
Averaged activity in region of low partial volume loss (Planes 58 to 67) (kBq/ml)	2895.02	25039.19
% decrease due to partial volume loss	**68%**	**54%**

The middle portion of the narrow end of the glass Pasteur pipette is a region with high partial volume loss and corresponds to planes 7 to 16 in the activity profile (Figure 8B). The wide end of the glass Pasteur pipette is a large volume with minimal partial volume loss and corresponds to planes 58 to 67 in the activity profile (Figure 8B). The percentage of partial volume losses are more pronounced when the overall activity in the phantom is low, i.e. 68% loss in the 1 MBq-phantom compared to 54% loss in the 8 MBq-phantom.

In summary, the phantom modeled in dimension and activity on the actual bone uptake data in our [^18^F]fluoride microPET, suggests that spillover effect and partial volume loss are a likely major cause why the expected biological variations in our animal models of altered bone turnover could not be adequately detected.

### Conclusion

Our study shows that, like in other organ systems, there is a significant expression of the TSPO in bone tissue and that the expression levels of TSPO relate to active tissue changes. The exact role of the TSPO in physiological and pathological bone turnover remains to be studied. However, there are now a number of pharmacologically active ligands for the TSPO available. These are so far used for other purposes such as anxiolysis [39], [40], and their effects on bone are not known. The turnover dependent presence of TSPO in bone provides a rational to study the TSPO as potential therapeutic target in diseased bone.

While *in vivo* molecular imaging of bone to measure the cellular correlates of bone pathology is desirable, the available instrumentation and radioligands require further refinement to undertake systematic studies in experimental animal models.

## Supporting Information

Table S1 **PCR primers.** Primer sequences for PCR amplification of TSPO, ALP, TRAP and GAPDH genes.
(DOC)
