# Correction: The 18 kDa Translocator Protein (Peripheral Benzodiazepine Receptor) Expression in the Bone of Normal, Osteoprotegerin or Low Calcium Diet Treated Mice

**DOI:** 10.1371/annotation/a7585d21-c427-498e-936e-5a548fe113bf

**Published:** 2012-10-08

**Authors:** Winnie Wai-Ying Kam, Steven R. Meikle, Hong Zhou, Yu Zheng, Julie M. Blair, Marcus Seibel, Colin R. Dunstan, Richard B. Banati

Four authors were erroneously excluded from the author list.

The correct byline is: Winnie Wai-Ying Kam^1,2,4*^, Steven R. Meikle^1,2^, Hong Zhou^3^, Yu Zheng^3^, Julie M. Blair^3,6^, Marcus Seibel^3^, Colin R. Dunstan^3,5^, Richard B. Banati^1,2,4^.

The correct affiliations are:

1 Discipline of Medical Radiation Sciences, Faculty of Health Sciences, University of Sydney, Cumberland, New South Wales, Australia,

2 Ramaciotti Imaging Center, Brain and Mind Research Institute, University of Sydney, Camperdown, New South Wales, Australia,

3 Bone Research Program, Anzac Research Institute, University of Sydney, Concord, New South Wales, Australia,

4 Australian Nuclear Science and Technology Organisation, Lucas Heights, New South Wales, Australia,

5 School of Aerospace, Mechanical and Mechatronic Engineering, University of Sydney, Camperdown, New South Wales, Australia,

6 Department of Medical Affairs, Amgen Australia Ptv. Ltd., Macquarie Park, New South Wales, Australia.

The correct citation is: Kam WW-Y, Meikle SR, Zhou H, Zheng Y, Blair JM, et al. (2012) The 18 kDa Translocator Protein (Peripheral Benzodiazepine Receptor) Expression in the Bone of Normal, Osteoprotegerin or Low Calcium Diet Treated Mice. PLoS ONE 7(1): e30623. doi:10.1371/journal.pone.0030623

The correct author contributions are: Conceived and designed the experiments: WW-YK SRM MS CRD RBB. Performed the experiments: WW-YK SRM HZ YZ JMB CRD. Analyzed the data: WW-YK SRM HZ YZ JMB CRD RBB. Contributed reagents/materials/analysis tools: SRM HZ YZ JMB CRD RBB. Wrote the paper: WW-YK. 

