# Supplementary material for: The 18 kDa Translocator Protein (Peripheral Benzodiazepine Receptor) Expression in the Bone of Normal, Osteoprotegerin or Low Calcium Diet Treated Mice
Source: PLoS One. 2012 Jan 25;7(1):e30623. doi: 10.1371/journal.pone.0030623 (PMC3266288; doi:10.1371/journal.pone.0030623)
Supplement: Table S1 — PCR primers. Primer sequences for PCR amplification of TSPO, ALP, TRAP and GAPDH genes. (DOC) [file pone.0030623.s001.doc]

**Supporting information**

**Table S1. PCR primers**

| **Gene** | **Reference** | **Forward Primer** | **Reverse Primer** |
| --- | --- | --- | --- |
| TSPO | n/a | 5’-GGGAGCCTACTTTGTGCGTGG-3’ | 5’-CAGGTAAGGATACAGCAAGCGGG-3’ |
| ALP | [30] | 5’-CTTGACTGTGGTTACTGCTG-3’ | 5’-GAGCGTAATCTACCATGGAG-3’ |
| TRAP | [31] | 5’-CGACCATTGTTAGCCACATACG-3’ | 5’-TCGTCCTGAAGATACTGCAGGTT-3’ |
| GAPDH | [32] | 5’-CCATGGAGAAGGCTGGGG-3’ | 5’-CAAAGTTGTCATGGATGACC-3’ |
